# Iron Deficiency in Heart Failure: From ESC Guidelines to Clinical Practice at a Romanian Hospital

**DOI:** 10.3390/biomedicines13092296

**Published:** 2025-09-19

**Authors:** Oana Sirbu, Andreea Tirnoveanu, Raluca Ecaterina Haliga, Victorita Sorodoc, Miruna Sava, Cristina Bologa, Ovidiu Rusalim Petris, Bianca Codrina Morarasu, Alexandra Diana Diaconu, Alexandr Ceasovschih, Catalina Lionte, Paula Cristina Morariu, Branco Adrian Morariu, Cristian Statescu, Radu Andy Sascau, Mariana Floria, Laurentiu Sorodoc

**Affiliations:** 1 Faculty of Medicine, Grigore T. Popa University of Medicine and Pharmacy, 16 University Street, 700115 Iasi, Romania; 2 Department of Internal Medicine, St. Spiridon Clinical Emergency Hospital, 700111 Iasi, Romania; 3 Department of Cardiology, Institute of Cardiovascular Diseases “Dr. George I.M. Georgescu”, 700503 Iasi, Romania

**Keywords:** iron deficiency, heart failure, anemia, iron supplementation, intravenous ferric carboxymaltose

## Abstract

**Background:** Iron deficiency (ID) is a frequent comorbidity in heart failure (HF), associated with reduced functional capacity and poor prognosis. Although the European Society of Cardiology (ESC) guidelines recommend systematic screening and intravenous iron supplementation (IS), adherence in clinical practice remains limited. This observational study aimed to evaluate how these recommendations are implemented into practice. **Methods:** We performed a retrospective study including 4348 patients hospitalized with HF (NYHA II-IV) in a tertiary internal medicine clinic in Eastern Europe between January 2018 and September 2022. Demographic data, comorbidities, laboratory parameters, echocardiographic findings were collected from electronic medical records. IS was defined as serum ferritin < 100 ng/mL. **Results:** Among HF patients, 2547 (58.7%) were screened for ID, and 1091 (42.8%) had absolute deficiency. Only 278 patients (25.5%) received intravenous ferric carbodymaltose. Treated patients were predominantly elderly (70.1% ≥ 70 years), female (60.4%), and often had ischemic or valvular disease. Patients receiving intravenous IS showed higher NT-proBNP and troponin levels. A progressive increase in IS use was observed during the study period, with a temporary decline during the COVID-19 pandemic. **Conclusions:** Despite relatively high screening rates, only one-quarter of HF patients with confirmed ID received intravenous IS. These findings highlight persistent gaps between guidelines and clinical practice, emphasizing the need for improved awareness and implementation of ESC recommendations to optimize outcomes in HF patients with ID.

## 1. Introduction

Heart failure (HF) is a major global health issue, significantly impacting patients’ quality of life and survival. It occurs when the heart is unable to pump enough blood to meet the body’s needs, leading to symptoms such as fatigue, breathlessness, and fluid retention. It affects millions of people worldwide and represents a major cause of hospitalization, morbidity, and mortality. Despite advances in pharmacological and device-based therapies, HF remains associated with high rates of hospitalization and adverse clinical outcomes [1].

Iron deficiency (ID) is a highly prevalent and clinically significant comorbidity in HF, affecting up to 50% of patients with stable chronic HF and 80% of those hospitalized with acute decompensated HF. It is associated with worse symptoms, reduced exercise capacity, and poorer prognosis, independent of anemia, emphasizing the need for systematic screening and targeted management [2,3]. The mechanisms underlying ID and HF are multifactorial, including inadequate dietary intake, gastrointestinal congestion, impaired absorption, altered distribution, increased losses, and chronic low-grade inflammation. Inflammatory signaling, particularly via IL-6-mediated activation of the JAK-STAT3 pathway, stimulates hepatic hepcidin production. Hepcidin, a central regulator of iron homeostasis, binds ferroportin, triggering its degradation and thereby reducing both gastrointestinal iron absorption and mobilization from storage sites such as macrophages and hepatocytes. This leads to functional ID, characterized by iron sequestration and limited availability for erythropoiesis and cellular functions. Iron losses are further amplified by the frequent use of antiplatelet and anticoagulant therapies in HF patients, contributing to the overall burden of deficiency [2,3,4].

The presence of ID in HF patients is associated with adverse clinical outcomes, including reduced exercise capacity, early muscle fatigue, and impaired peak oxygen consumption, reflecting iron’s essential role in mitochondrial function and skeletal muscle metabolism. ID also increases the risk of HF-related hospitalizations and is an independent predictor of all-cause and cardiovascular mortality. Given its prognostic significance, international guidelines recommend routine screening. The European Society of Cardiology (ESC) guidelines define absolute ID as ferritin <100 ng/mL and functional ID as ferritin 100–299 ng/mL with transferrin saturation (TSAT) <20% [5].

Intravenous iron, particularly ferric carboxymaltose (FCM), has demonstrated consistent benefits in HF, improving functional capacity, alleviating symptoms, enhancing quality of life, and reducing hospitalizations, as shown in FAIR-HF, CONFIRM-HF, and AFFIRM-AHF trials. In contrast, oral iron is limited by poor absorption, intolerance, and persistent hepcidin elevation [5]. Despite strong evidence, intravenous iron remains underutilized in clinical practice due to low physician awareness, insufficient routine assessment of iron status, economic constraints, restricted availability, and diagnostic challenges such as inconsistent measurement of TSAT. These barriers contribute to underdiagnosis and delayed treatment, adversely affecting patient outcomes [5].

Given the high prevalence of ID in HF and the notable gap between guideline recommendations and current clinical practices, this study aims to evaluate the real-world implementation of the ESC guidelines for the diagnosis and management of ID in HF patients in Eastern Europe. The primary objective of this study was to assess the current practice of iron status screening among HF patients within an internal medicine clinic in this region and to determine the prevalence of ID in this population. The secondary aim was to evaluate the appropriateness and utilization of iron supplementation (IS), with intravenous therapy, in patients diagnosed with ID. Ultimately, the findings from this research aim to offer valuable insights that could shape future efforts to enhance the detection and treatment of ID in HF, leading to improved patient outcomes and a reduction in HF-related hospitalizations and mortality.

## 2. Materials and Methods

### 2.1. Design of the Study

This study was a retrospective study, performed on a group of patients with HF, hospitalized in the Medical Clinic of the Saint Spiridon Emergency Hospital in Iasi, Romania, between January 2018 to September 2022, for the evaluation and management of their conditions.

### 2.2. Data Collection

Retrospectively, we selected the patients with a diagnosis of HF according to the International Classification of Diseases using the codes: I50.0, I150.1, and I50.9. We gathered various patient data from hospital electronic medical records and individual patient observation sheets, including demographic characteristics, personal and medical history, cardiovascular risk factors, laboratory results, echocardiography parameters, and treatment administered. Laboratory tests for iron status and other relevant parameters were recorded at the time of hospital admission.

### 2.3. Assessment of ID and Anemia in HF

In patients with HF, ID was defined as an SF level below 100 ng/mL, absolute ID [4]. Although the guideline [6] also classifies ID as an SF level between 100–299 ng/mL with TSAT below 20% (functional ID), we chose to exclude patients with functional ID due to the inconsistent availability of transferrin saturation in our hospital. Therefore, our focus was solely on patients with absolute ID, defined as an SF value under 100 ng/mL. Anemia was defined based on World Health Organization recommendations as a hemoglobin (Hb) level below 12.0 g/dL in women and below 13.0 g/dL in men [7]. Intravenous iron therapy was administered during hospitalization according to calculated iron requirements and clinical judgment. Patients received one or two vials of FCM, depending on the individual ID and the treating physician’s decision. In some cases, oral IS was prescribed at discharge if the calculated iron requirement had not been fully met during hospitalization.

### 2.4. Statistical Analysis

The data were centralized in SPSS 18.0 databases and analyzed using applicable statistical functions. The statistical analysis utilized both descriptive and analytical approaches. Descriptive analysis was carried out with ANOVA test, which revealed the qualitative aspects of the group, aiming at the relationship of interdependence between variables, at the 95% significance level. The Kurtosis test with values ranged between –2 and 2, tested the normality of the series (continuous variable). In analyzing the significant difference between two or more groups, according to the distribution of the series of values, at the 95% significance level, the following tests were applied for quantitative variables: t-Student test (a parametric test which compares the mean values recorded in 2 groups with normal distributions) and ANOVA Bonferroni post hoc test (a parametric test comparing the mean values recorded in 3 or more groups with normal distributions, involving a sequence of t-tests where the significance level is divided by the number of comparisons). The c2 test is a non-parametric test that compares 2 or more frequency distributions from the same population, and was applied when the expected events are excluded. The Kruskal–Wallis correlation compares ordinal variables from 3 or more groups. The Pearson correlation coefficient (r) represents the correlation of 2 variables in the same group, the direct/indirect correlation being given by the sign of the coefficient.

## 3. Results

**Figure 1:**
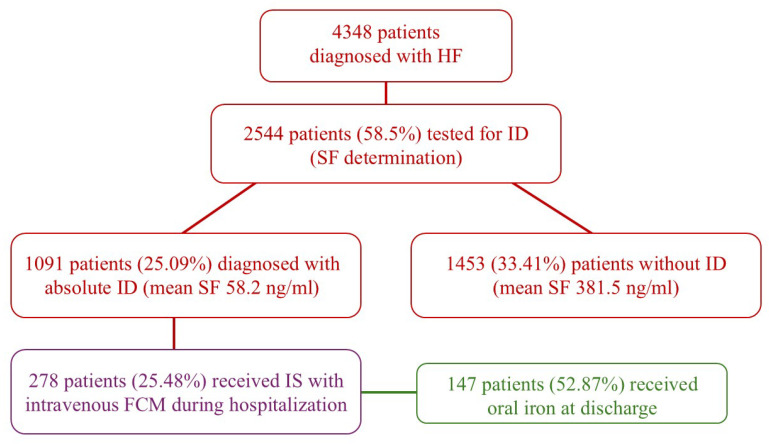
Diagram of the study design. FCM ferric carboxymaltose; HF heart failure; ID iron deficiency; SF serum ferritin.

During the inclusion period, a total of 4348 patients diagnosed with HF were treated in our clinic, as shown in Figure 1. Of the total, 2547 patients (58.5%) were assessed for ID using ferritin. Investigations revealed that 1091 (42.83%) of these patients had absolute ID (SF < 100 ng/mL), with an average SF level of 58.2 ng/mL. Of those diagnosed with absolute ID, 278 patients (25.48%) received IS with intravenous FCM.

The patients receiving IS with FCM were assessed to understand the reasons why medical practitioners choose only these patients who received the recommended treatment. General characteristics of patients are detailed in Table 1. Based on the comparative analysis of patients stratified by Hb levels, several significant associations were observed. Patients with Hb ≤ 12 g/dL were significantly more likely to be classified in higher NYHA classes (*p* = 0.045), and to have a history of myocardial infarction (*p* = 0.036), hypertension (*p* = 0.045), valvular disease (*p* = 0.038), and bleeding events (*p* = 0.027).

Table 2 presents the characteristics of patients with reduced and preserved ejection fraction. The discrepancy in patient numbers reflects the lack of complete echocardiographic measurements in all patients, who were therefore excluded from this analysis. The univariate analysis revealed several significant associations between left ventricle ejection fraction (LVEF) below 40% and various demographic and clinical parameters. Male patients exhibited a higher prevalence of reduced EF compared to females (52.2% vs. 35.1%, *p* = 0.036). Patients classified as New York Heart Association (NYHA) Class III were more likely to have reduced EF (67.4% vs. 34.2%, *p* = 0.001). A history of myocardial infarction (78.3% vs. 54.1%, *p* = 0.004), hypertension (56.5% vs. 73.0%, *p* = 0.035), acute myocardial infarction (15.2% vs. 3.6%, *p* = 0.015), stroke (21.7% vs. 8.1%, *p* = 0.020), and valvulopathy (82.6% vs. 60.4%, *p* = 0.005) were all significantly associated with a higher prevalence of reduced EF.

The biological and echocardiographic parameters of patients with an LVEF below 40% are detailed in Table 3. Patients with LVEF below 40% also showed significantly higher levels of Hb (9.97 vs. 8.18; *p* = 0.001), hematocrit (31.07 vs. 25.86; *p* = 0.001), and troponin (163.31 vs. 93.22; *p* = 0.048). Additionally, echocardiographic parameters were notably elevated in these patients, with significantly higher values for left ventricular and diastolic diameter (LVSD: 53.15 vs. 40.10; *p* = 0.002), left ventricular end-systolic diameter (LVESD: 41.09 vs. 27.28; *p* = 0.001), and right ventricular end-diastolic diameter (RVEDD: 26.67 vs. 21.63; *p* = 0.05).

Regarding the treatment recommended to patients in the study group, according to Table 4, Furosemide was significantly more prescribed to patients with an LVEF of 40% or less compared to those with an LVEF greater than 40% (50.0% vs. 32.4%, *p* = 0.030). In contrast, other treatments, including Aspirin, Clopidogrel, anticoagulants, beta-blockers, and ACE inhibitors/ARBs, were administered uniformly across both LVEF groups, suggesting that these therapies are not influenced by the severity of cardiac dysfunction.

**Figure 2:**
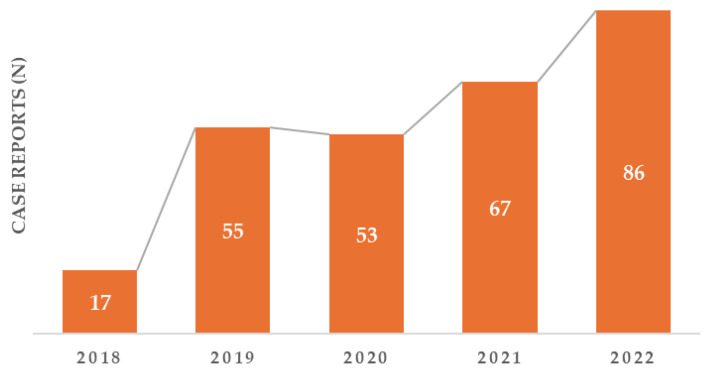
Yearly distribution of patients who received treatment during the study period.

Of the 1091 patients diagnosed with HF and absolute ID (SF <100 ng/mL), 278 patients (25.48%) were treated with intravenous FCM. As illustrated in Figure 2, there has been a steady increase over the years in the treatment of patients diagnosed with HF and absolute ID.

Table 5 presents baseline clinical and laboratory characteristics of patients treated with FCM, stratified by dose (two vials, n = 166; one vial, n = 112). The distribution of NYHA class, cardiac disease history, and most laboratory indices did not differ significantly between groups. A history of aspirin use was higher in the two-vial group (*p* = 0.023). Hematological and iron parameters were comparable, but patients in the two-vial group had significantly higher NT-proBNP (*p* = 0.028) and troponin levels (*p* = 0.001). Other laboratories or echocardiographic values, including BNP or LVEF, showed no significant differences.

According to Table 6, in the group of patients prescribed oral IS upon discharge, a significant proportion demonstrated advanced HF, with over 40% classified as NYHA Class III-IV (*p* = 0.349). Additionally, 23.1% of these patients presented with bleeding episodes (*p* = 0.003). Comorbidities were prevalent, including a history of hypertension (58.5%, *p* = 0.028), myocardial infarction (11.6%, *p* = 0.014), and stroke (7.5%, *p* = 0.013).

In correlation with the data presented in Table 7, patients who were discharged with oral IS exhibited significantly lower levels of Hb (7.88, *p* = 0.002), hematocrit (25.45, *p* = 0.02), ferritin (44.57, *p* < 0.001), C-reactive protein (2.81, *p* = 0.029), and troponin (60.18, *p* = 0.011).

## 4. Discussion

ID is a common comorbidity in HF patients, with studies indicating a prevalence of around 50% in those with stable chronic HF and up to 80% in those hospitalized with AHF [8]. ID is associated with reduced functional capacity, poorer QoL, and worse prognosis [6,8,9,10]. Randomized clinical trials have shown that intravenous FCM can significantly improve functional capacity, enhance QoL, and reduce the risk of hospitalization in HF patients [6,10,11,12]. Unfortunately, there is currently limited data on the global use of intravenous IS in HF. A few studies indicate that its implementation remains low, ranging between 3% and 24% [13,14].

The 2016 ESC Guidelines for the diagnosis and treatment of acute and chronic HF recommend intravenous FCM for patients with symptomatic HF, reduced LVEF, and ID [10]. These guidelines also highlight the importance of regularly monitoring patients for ID and anemia.

After five years, the 2021 ESC Guidelines for the diagnosis and treatment of acute and chronic HF were updated to highlight the importance of screening for ID and anemia in all HF patients, not just those with reduced LVEF. This screening should include checking a full blood count, SF concentration, and TSAT (recommendation class I, evidence level C).

Despite clear guidelines, clinical practice often falls short of these recommendations. The systematic assessment of ID is frequently overlooked or inconsistently applied, either due to limited resources, inadequate knowledge, or possibly disinterest among healthcare providers. This gap between guidelines and practice can result in the underdiagnosis and insufficient treatment of HF-related comorbidities, including the delayed detection of ID, which adversely affects patient outcomes and overall health.

For example, in a large study conducted between 2017 and 2018, which included 21496 patients with HF from the Swedish HF registry, only 27% of patients were tested for ID, and 20% of patients received intravenous FCM [12]. These figures are low considering the approximately 50% prevalence of ID in stable HF and even higher in patients with AHF [8,12]. Similarly, a multicenter study conducted in Spain between 2014 and 2019 on a total of 3555 patients with AHF found that 47.8% of AHF patients were screened for ID during hospitalization [15]. Another study, the OFICSel study, carried out by a team of 300 cardiologists from the French HF Group across 13 regions in France, surveyed 2822 patients between March and June 2017. The study found that ID was assessed in only 38.1% of the patients [16].

The study conducted by our team collected data that reflects the pattern of HF patients in Eastern Europe. The findings showed that out of 4348 HF patients hospitalized in our clinic, 2547 (58.5%) were tested for ID, a higher testing rate compared to previous studies. Out of the patients tested in our clinic, 1091 (42.83%) were diagnosed with absolute ID (SF < 100 ng/mL), with an average SF level of 38.77 ng/mL. Despite these diagnoses, only a small number of 278 patients (25.48%) received IS with intravenous FCM.

Our study revealed a higher prevalence of HF patients diagnosed with ID among women (60.4%), similar to findings in the OFICSel study [16] and the study conducted in Spain [15]. While the OFICSel study reported a mean age of 66 years, the patients in our study had an older average age of 75 years, approximately the same as in the study group from Spain, where the average age was 74 years [15]. In our study, the highest proportion of cases was in the 80–89 age group (34.9%). Additionally, 70.1% of our patients were over 70 years old.

The results of our study align with the findings from similar research [12,15,16], indicating that patients hospitalized for HF are typically elderly, with a significant majority having preserved LVEF, with 70% of the patients presenting a LVEF > 40% in our study. The baseline characteristics of the patients in our study also resemble those of previous cohorts studied in Europe [16,17,18,19,20,21].

Several studies have indicated that ID in patients with HF may result from gastrointestinal bleeding or be worsened by using anticoagulants or antiplatelet medications [22,23,24,25,26,27]. Patients with HF are more prone to gastrointestinal injuries than the general population due to several risk factors, including gastrointestinal bleeding, advanced age, other comorbidities and the use of multiple medications. Antiplatelet drugs [25,26,28] and anticoagulants are known to increase the risk of gastrointestinal bleeding, especially when used together [23,24]. As a result, HF patients often have a weakened gastric lining, and the use of these medications to manage other health conditions makes them more vulnerable to gastrointestinal injury [22]. This means that chronic gastrointestinal bleeding is also closely linked to the development of ID. In our study, 41.4% of the patients were receiving oral anticoagulants, with 75.7% of them being over 70 years of age (*p* = 0.06). Additionally, 7.2% of the patients were treated with the antiplatelet drugs, with 80% of these patients also being over 70 years old (*p* = 0.05). These findings came close to achieving the statistical significance observed in earlier studies.

Although the guidelines do not recommend intravenous iron administration for patients with HF and preserved ejection fraction, clinicians have still used this approach, and we aimed to understand their motivation behind this practice. When stratifying patients by EF, we observed a substantial proportion of those with preserved EF receiving treatment, a finding consistent with existing data, including reports from the Swedish HF Registry [29] or other publications [30,31] and meta-analyses [32]. These findings highlight the importance of screening for anemia and ID in all patients with HF.

Also, these patients had a lower Hb level, lower NT-proBNP, troponin levels, and the measurement of ventricular chambers was lower, indicating that these patients had associated other conditions that could lead to anemia and were treated with FCM. Findings indicate that in HF with preserved ejection fraction (HFpEF), NT-proBNP levels are typically lower than in HF with reduced ejection fraction (HFrEF), partly due to less pronounced myocardial stretch and troponin levels [33,34]. It has also been demonstrated that HFpEF patients typically have smaller LV volumes and thicker walls compared to HFrEF [35].

The progression of HF is a complex process often accompanied by a significant number of comorbidities [36,37,38]. The study identified several comorbid conditions in patients with HF and ID, including AFib, HT, and CAD, similar to those published by the Swedish HF Registry [29]. Additionally, conditions such as valvular heart disease, a history of acute myocardial infarction, and neoplasms were also associated with these patients.

Our results indicate that the ESC recommendations [6] to screen all HF patients for ID and anemia, as well as to provide intravenous FCM to those diagnosed, are still not being fully implemented. Several factors may explain the lack of ID screening. First, the study began just two years after the 2016 ESC Guidelines for the diagnosis and treatment of acute and chronic HF were published [10], suggesting that more time may be needed for these guidelines to be integrated into medical practice. Despite this, there has been a gradual increase over the years in the number of patients who received IS with intravenous FCM. For example, only 17 patients received IS in 2018, while that number rose to 86 in 2022. However, there was a notable exception in 2020, when the number of patients receiving IS decreased. This decline can be attributed to the onset of the COVID-19 pandemic caused by SARS-CoV-2. During this time, our clinic served as a primary care center for critically ill patients, which limited the hospitalization of individuals with chronic cardiac conditions, as medical care was primarily directed toward those infected with the virus.

We tried to identify the patients who also received oral IS after discharge, and it was observed that this decision of the practitioners was independent of the level of Hb or the bleeding; patients with higher troponin levels and lower EF were preferred to receive a supplemental oral iron treatment. Current clinical guidelines (ESC 2021, AHA 2022) recommend ID screening in all HF patients, irrespective of Hb levels, troponin values, or EF. Iron supplementation in HF is determined based on SF and TSAT levels rather than Hb concentration [6,39].

In medical practice, we frequently encounter logistical issues, such as the temporary unavailability of certain reagents and financial constraints, which can lead to patients with ID remaining undiagnosed. Intravenous IS are indeed more expensive than oral preparations, which may explain the limited recommendations of IS for patients with ID.

The main limitation of the study is the impossibility of evaluating the functional ID in the selected group of patients and including these patients in our analysis. Even though the actual guidelines state that all patients with HF should be investigated for ID, the availability of transferrin saturation in the hospital is lacking due to economic reasons. In addition, due to the retrospective nature of the study and variability in clinical records, the exact degree of decompensation of HF at admission could not be consistently determined for all patients. Another limitation is the superposition of the pandemic period on our study timeline. For many months, our clinic treated only COVID-19 patients, so the results could be influenced by these aspects, mostly because the focus of the medical practitioners was mostly on treating the infection, and ID could have been disregarded in patients that associated also had HF.

## 5. Conclusions

Iron deficiency is a prevalent comorbidity among HF patients, significantly affecting their functional capacity, QoL, and overall prognosis. Despite established guidelines recommending routine screening and treatment for ID in HF patients, the implementation of these recommendations remains inadequate. Our study demonstrated a higher testing rate for ID compared to previous research; however, only a small percentage of diagnosed patients received intravenous IS. Factors contributing to the underdiagnosis and undertreatment of ID include limited resources, inadequate provider knowledge, and logistical challenges within the healthcare system, exacerbated by the impact of the COVID-19 pandemic. Given the association between ID and adverse outcomes in HF, it is crucial to enhance awareness and adherence to screening and treatment guidelines among healthcare professionals to improve patient outcomes. Continued efforts are needed to address barriers to effective ID management in HF patients, ensuring that those at risk receive timely and appropriate care.

## Figures and Tables

**Table 1:** General characteristics of patients according to hemoglobin level.

Parameter	Hemoglobin Level			*p* Value for Chi- Square Test/ Fisher’s Exact Test
	≤12 g/dL (n = 263)	>12 g/dL (n = 15)	Study Group (n = 278)	
**Demographic Characteristic**				
Female Sex	165 (62.7%)	3 (20.0%)	168 (60.4%)	**0.001**
Age ≥70 years	189 (71.9%)	6 (40.0%)	195 (70.1%)	**0.012**
**NYHA Class**				
II	71 (27.0%)	7 (46.7%)	78 (28.1%)	**0.045**
III	92 (35.0%)	7 (46.7%)	99 (35.6%)	
IV	9 (3.4%)	0 (0.0%)	9 (3.2%)	
**Personal Medical History**				
Aortic Stenosis	37 (14.1%)	3 (20.0%)	40 (14.4%)	0.369
Myocardial Infarction	104 (39.5%)	10 (66.7%)	114 (41.0%)	**0.036**
Chronic Coronary Syndrome	88 (33.5%)	4 (28.7%)	92 (33.1%)	0.408
Hypertension	172 (65.4%)	6 (40.0%)	178 (64.0%)	**0.045**
Atrial Fibrillation	125 (47.5%)	6 (40.0%)	131 (47.1%)	0.383
Acute Myocardial Infarction	20 (7.6%)	2 (13.3%)	22 (7.9%)	0.336
Stroke	30 (11.4%)	3 (20.0%)	33 (11.9%)	0.257
Diabetes Mellitus	84 (31.9%)	4 (26.7%)	88 (31.7%)	0.457
Valvular Disease	122 (46.4%)	11 (73.3%)	133 (47.8%)	**0.038**
Asthma	6 (2.3%)	0 (0.0%)	6 (2.2%)	0.715
COPD	7 (2.7%)	1 (6.7%)	8 (2.9%)	0.362
Malignancy	77 (29.3%)	4 (9.7%)	78 (28.1%)	**0.045**
Bleeding	85 (32.3%)	1 (6.7%)	86 (30.9%)	**0.027**
**Treatment**				
Aspirin	27 (10.3%)	1 (6.7%)	28 (10.1%)	0.541
Clopidogrel	18 (7.2%)	1 (6.7%)	20 (7.2%)	0.706
Oral Anticoagulant (OAC)	109 (41.4%)	6 (40.0%)	115 (41.4%)	0.568
Oral Iron at Discharge	127 (48.3%)	4 (26.7%)	131 (47.1%)	0.084
Beta-Blockers	130 (49.4%)	9 (60.0%)	139 (50.0%)	0.298
Calcium Channel Blockers (CCB)	39(14.8%)	2 (13.3%)	41 (14.7%)	0.615
ACEi/ARB	79 (30.0%)	3 (20.0%)	82 (29.5%)	0.305
Furosemide	94 (35.7%)	5 (33.3%)	99 (35.6%)	0.544
Spironolactone	74 (28.1%)	5 (33.3%)	79 (28.4%)	0.430

**Table 2:** General characteristics of patients according to left ventricular ejection fraction.

Parameter	Ejection Fraction			*p*-Value for Chi- Square Test/ Likelihood Ratio
	≤40% (n = 46)	>40% (n = 111)	Study Group (n = 157)	
**Demographic characteristic**				
Male sex	24 (52.2%)	39 (35.1%)	63 (40.1%)	**0.036**
Age ≥70 years	32 (69.6%)	86 (77.5%)	118 (75.2%)	0.199
**NYHA Class**				
II	9 (19.6%)	40 (36.0%)	49 (31.2%)	**0.001**
III	31 (67.4%)	38 (34.2%)	69 (43.9%)	
IV	3 (6.5%)	3 (2.7%)	6 (3.8%)	
**Personal Medical History**				
Aortic Stenosis	11 (23.9%)	21 (18.9%)	32 (20.4%)	0.485
Myocardial Infarction	36 (78.3%)	60 (54.1%)	96 (61.1%)	**0.004**
Chronic Coronary Syndrome	14 (30.4%)	38 (34.2%)	52 (33.1%)	0.395
Hypertension	26 (56.5%)	81 (73.0%)	107 (68.2%)	**0.035**
Atrial fibrillation	27 (58.7%)	55 (49.5%)	82 (52.2%)	0.193
Acute Myocardial Infarction	7 (15.2%)	4 (3.6%)	11 (7.0%)	**0.015**
Stroke	10 (21.7%)	9 (8.1%)	19 (12.1%)	**0.020**
Diabetes Mellitus	13 (28.3%)	29 (26.1%)	42 (26.8%)	0.464
Valvular Disease	38 (82.6%)	67 (60.4%)	105 (66.9%)	**0.005**
Asthma	1 (2.2%)	2 (1.8%)	3 (1.9%)	0.649
COPD	2 (4.3%)	3 (2.7%)	5 (3.2%)	0.458
Malignancy	9 (19.6%)	32 (28.8%)	41 (26.1%)	0.158
Bleeding	8 (17.4%)	31 (27.9%)	39 (24.8%)	0.116

**Table 3:** Laboratory and echocardiographic parameters of patients according to left ventricular ejection fraction.

Parameter	Ejection Fraction			*p* Value for t-Student Test
	EF ≤ 40% (n = 46)	EF > 40% (n = 111)	Study Group (n = 157)	
**Laboratory Parameter** **(Mean ± SD)**				
Hemoglobin (g/dL)	9.97 ± 2.72	8.18 ± 2.48	8.71 ± 2.72	**0.001**
Hematocrit (%)	31.07 ± 7.88	25.86 ± 8.37	27.38 ± 8.54	**0.001**
White blood cells (μL)	6557 ± 4360	7625 ± 3379	7898 ± 3703	0.152
Iron (μg/dL)	48.04 ± 7.38	35.63 ± 3.67	39.27 ± 42.62	0.096
Ferritin (ng/mL)	57.06 ± 3.97	31.19 ± 4.47	38.77 ± 6.07	0.653
Uric acid (mg/dL)	5.02 ± 4.33	4.26 ± 3.51	4.48 ± 3.77	0.265
Blood glucose (mg/dL)	117.15 ± 64.81	113.66 ± 57.01	114.68 ± 59.21	0.738
Total cholesterol (mg/dL)	108.51 ± 72.63	96.77 ± 73.46	100.15 ± 73.18	0.365
HDL-C (mg/dL)	23.87 ± 16.80	27.01 ± 21.80	26.09 ± 20.46	0.383
LDL-C (mg/dL)	62.28 ± 60.27	55.52 ± 50.04	57.50 ± 53.13	0.470
VEM (fL)	80.95 ± 18.71	78.38 ± 22.10	79.14 ± 21.13	0.490
HEM (pg)	20.51 ± 12.29	20.52 ± 12.66	20.52 ± 12.51	0.995
CHEM (g/dL)	29.87 ± 7.66	29.36 ± 6.46	29.51 ± 6.82	0.669
GR (×10^3^/μL)	3617 ± 962	3247 ± 981	3356 ± 987	**0.033**
RA (mmol/L)	22.08 ± 7.76	20.84 ± 8.68	21.20 ± 9.16	0.453
Urea (mg/dL)	60.03 ± 31.04	51.94 ± 26.74	54.31 ± 28.21	0.102
Creatinine (mg/dL)	1.16 ± 0.44	1.02 ± 0.44	1.06 ± 0.46	0.086
Creatinine clearance (mL/min)	63.65 ± 25.06	67.09 ± 25.36	66.08 ± 25.2	0.439
CRP (mg/L)	2.70 ± 4.41	4.03 ± 1.22	3.64 ± 0.88	0.491
NTproBNP (pg/mL)	4278 ± 1250	2034 ± 350	2982 ± 455	**0.001**
BNP (pg/mL)	577.65 ± 144.38	464.83 ± 184.48	514.45 ± 170.33	0.284
Troponin (ng/L)	163.31 ± 132.80	93.22 ± 52.24	113.75 ± 53.46	**0.048**
**Cardiovascular Parameter**				
LVEDD (mm)	53.15 ± 23.55	40.10 ± 23.20	43.92 ± 23.98	**0.002**
LVESD (mm)	41.09 ± 21.64	27.28 ± 20.18	31.32 ± 21.49	**0.001**
RVEDD (mm)	26.67 ± 15.46	21.63 ± 14.79	23.13 ± 15.12	**0.050**
IVS (mm)	10.61 ± 4.10	9.60 ± 5.31	9.89 ± 5.00	0.256
LVPW (mm)	10.20 ± 3.76	8.70 ± 5.66	9.13 ± 5.22	0.105

**Table 4:** Baseline medications according to left ventricular ejection fraction.

Parameter	Ejection Fraction			*p*-Value for Chi^2^ Test
	≤40% (n = 46)	>40% (n = 111)	Study Group (n = 157)	
**Treatment**				
Aspirin	6 (13.0%)	14 (12.6%)	20 (12.7%)	0.941
Clopidogrel	5 (10.9%)	9 (8.1%)	14 (8.9%)	0.587
Oral Anticoagulant (OAC)	21 (45.7%)	47 (42.3%)	68 (43.3%)	0.704
Oral Iron at Discharge	21 (45.7%)	60 (54.1%)	81(51.6%)	0.338
Beta-Blockers	25 (54.3%)	65 (58.6%)	90 (57.3%)	0.378
Calcium Channel Blockers (CCB)	8 (17.4%)	17 (15.3%)	25 (15.9%)	0.458
ACEi/ARB	17 (37.0%)	30 (27.0%)	47 (29.9%)	0.148
Furosemide	23 (50.0%)	36 (32.4%)	59 (37.6%)	**0.030**
Spironolactone	17 (37.0%)	30 (27.0%)	47 (29.9%)	0.148

**Table 5:** Baseline characteristics of patients treated with intravenous ferric carboxymaltose.

Parameter	Intravenous Ferric Carboxymaltose (FCM)		*p* Value for Chi- Square Test/ Likelihood Ratio
	2 Vials (n = 166)	1 Vials (n = 112)	
**NYHA Class**			
II	45 (29.0%)	31 (27.7%)	0.207
III	53 (34.2%)	43 (38.4%)	
IV	8 (5.2%)	1 (0.9%)	
**History of cardiac disease**			
Aortic stenosis	20 (12.9%)	19 (17.0%)	0.356
Myocardial Infarction	61 (39.4%)	50 (44.6%)	0.387
Malignacy	41 (26.5%)	34 (30.4%)	0.484
Bleeding	40 (25.8%)	42 (37.5%)	**0.042**
**Treatment**			
Aspirin	21 (13.5%)	6 (5.4%)	**0.023**
Clopidogrel	13 (8.4%)	5 (4.5%)	0.197
Oral Anticoagulant (OAC)	60 (38.7%)	47 (42.0%)	0.593
**Laboratory Parameter** **(Mean ± SD)**			
Hemoglobin (g/dL)	8.63 ± 3.23	8.11 ± 2.59	0.607
Hematocrit (%)	27.20 ± 7.49	26.53 ± 8.22	0.487
VEM (fL)	79.04 ± 22.53	80.41 ± 17.03	0.591
HEM (pg)	21.89 ± 12.40	22.62 ± 9.93	0.349
CHEM (g/dL)	29.13 ± 8.03	30.47 ± 4.46	0.113
Iron (μg/dL)	40.24 ± 4.15	39.60 ± 4.34	0.917
Ferritin (ng/mL)	58.73 ± 11.24	62.15 ± 9.87	0.124
NTproBNP (pg/mL)	2947 ± 511	1522 ± 282	**0.028**
Troponin (ng/L)	307.77 ± 187.81	8.00 ± 5.51	**0.001**
EF %	24.77 ± 1.91	26.71 ± 2.38	0.523
Weight (kg)	76.96 ± 24.77	69.60 ± 16.00	0.055

**Table 6:** Clinical characteristics of patients discharged with oral iron therapy.

Parameter	Oral Iron at Discharge			*p*-Value for Chi- Square Test Likelihood Ratio
	Yes (n = 147)	No (n = 131)	Study group (n = 278)	
**Demographic Characteristic**				
Female Sex	85 (57.8%)	83 (63.4%)	168 (60.4%)	0.206
Age ≥70 years	102 (69.4%)	93 (71.0%)	195 (70.1%)	0.437
**NYHA Class**				
II	35 (23.8%)	43 (32.8%)	78 (28.1%)	0.349
III	56 (38.1%)	43 (32.8%)	99 (35.6%)	
IV	4 (2.7%)	5 (3.8%)	9 (3.2%)	
**Personal Medical History**				
Aortic Stenosis	23 (15.6%)	17 (13.0%)	40 (14.4%)	0.526
Myocardial Infarction	58 (39.5%)	56 (42.7%)	114 (41.0%)	0.577
Chronic Coronary Syndrome	54 (36.7%)	38 (29.0%)	92 (33.1%)	0.108
Hypertension	86 (58.5%)	92 (70.2%)	178 (64.0%)	**0.028**
Atrial Fibrillation	66 (44.9%)	65 (49.6%)	131 (47.1%)	0.252
Acute Myocardial Infarction	17 (11.6%)	5 (3.8%)	22 (7.9%)	**0.014**
Stroke	11 (7.5%)	22 (16.8%)	33 (11.9%)	**0.013**
Diabetes Mellitus	46 (68.7%)	42 (32.1%)	88 (31.7%)	0.496
Valvular Disease	73 (49.7%)	60 (45.8%)	133 (47.8%)	0.301
Asthma	5 (3.4%)	1 (0.8%)	6 (2.2%)	0.136
COPD	6 (4.1%)	2 (1.5%)	8 (2.9%)	0.182
Malignancy	40 (27.2%)	38 (29.0%)	78 (28.1%)	0.739
Bleeding	34 (23.1%)	52 (39.7%)	86 (30.9%)	**0.003**

**Table 7:** Laboratory parameters of patients prescribed oral iron therapy at discharge.

Parameter	Oral Iron at Discharge			*p*-Value for t-Student Test
	Yes (n = 147)	No (n = 131)	Study Group (n = 278)	
**Laboratory Parameters** **(Mean ± SD)**				
Hemoglobin (g/dL)	7.88 ± 2.49	8.98 ± 3.24	8.71 ± 2.67	**0.002**
Hematocrit (%)	25.45 ± 7.60	27.66 ± 8.11	27.38 ± 8.54	**0.020**
White blood cells (μL)	7918 ± 2697	8161 ± 3458	8047 ± 3667	0.582
Iron (μg/dL)	34.04 ± 3.37	43.88 ± 4.57	39.24 ± 2.91	0.091
Ferritin (ng/mL)	44.57 ± 9.98	51.92 ± 7.67	48.03 ± 9.68	**<0.001**
Uric acid (mg/dL)	4.17 ± 3.85	3.67 ± 3.65	3.91 ± 3.75	0.279
Blood glucose (mg/dL)	118.17 ± 53.81	107.61 ± 64.01	112.58 ± 59.55	0.738
Total cholesterol (mg/dL)	135.75 ± 49.07	139.25 ± 46.92	137.45 ± 47.92	0.633
HDL-C (mg/dL)	37.28 ± 13.22	39.51 ± 14.20	38.35 ± 13.70	0.305
LDL-C (mg/dL)	84.97 ± 42.81	87.21 ± 38.29	86.04 ± 40.60	0.734
Sodium (mEq/L)	135.77 ± 17.54	134.30 ± 23.01	134.99 ± 20.59	0.553
Potassium (mEq/L)	4.43 ± 0.89	4.27 ± 1.06	4.35 ± 0.98	0.174
VEM (fL)	80.80 ± 17.22	78.94 ± 22.56	79.81 ± 20.22	0.445
HEM (pg)	21.46 ± 11.49	22.02 ± 11.35	21.75 ± 11.40	0.683
CHEM (g/dL)	29.94 ± 5.31	29.39 ± 7.69	29.65 ± 6.67	0.499
GR (×10^3^/μL)	3158 ± 897	3303 ± 1017	3235 ± 964	0.212
RA (mmol/L)	21.26 ± 8.58	19.35 ± 10.43	20.24 ± 9.64	0.104
Urea (mg/dL)	54.87 ± 32.54	57.46 ± 35.95	56.24 ± 34.35	0.532
Creatinine (mg/dL)	1.03 ± 0.44	1.07 ± 0.55	1.06 ± 0.50	0.608
Creatinine clearance (mL/min)	68.35 ± 24.79	66.81 ± 32.78	67.54 ± 29.25	0.663
CRP (mg/L)	2.81 ± 4.33	4.46 ± 0.97	3.68 ± 0.54	**0.029**
NTproBNP (pg/mL)	2345 ± 453	2238 ± 426	2288 ± 310	**0.046**
Troponin (ng/L)	60.18 ± 38.38	272.81 ± 195.51	172.25 ± 104.66	**0.011**
EF%	47.09 ± 11.67	44.41 ± 11.80	45.79 ± 11.77	0.155

## Data Availability

The original contributions presented in this study are included in the article. Further inquiries can be directed to the corresponding author(s).

## Abbreviations

The following abbreviations are used in this manuscript:

ID	Iron deficiency
HF	Heart failure
AHF	Acute heart failure
ESC	The European Society of Cardiology
SF	Serum ferritin
TSAT	Transferrin saturation
IS	Iron supplementation
FCM	Ferric carboxymaltose
LVEF	Left ventricular ejection fraction
QoL	Quality of life
NYHA	New York Heart Association
HFrEF	Reduced ejection fraction
HFmrEF	Mildly reduced ejection fraction
HFpEF	Preserved ejection fraction
IL-6	Interleukin-6
EF	Ejection fraction
LVSD	Left ventricular systolic diameter
SVSD	Left ventricular septal thickness
RVEDD	Right ventricular end-diastolic diameter
LVEDD	Left ventricular end-systolic diameter
